# Fake News or Weak Science? Visibility and Characterization of Antivaccine Webpages Returned by Google in Different Languages and Countries

**DOI:** 10.3389/fimmu.2018.01215

**Published:** 2018-06-05

**Authors:** Nadia Arif, Majed Al-Jefri, Isabella Harb Bizzi, Gianni Boitano Perano, Michel Goldman, Inam Haq, Kee Leng Chua, Manuela Mengozzi, Marie Neunez, Helen Smith, Pietro Ghezzi

**Affiliations:** ^1^Brighton and Sussex Medical School, Falmer, United Kingdom; ^2^School of Computing, Engineering and Mathematics, University of Brighton, Brighton, United Kingdom; ^3^Universidade Federal do Rio Grande do Sul, Porto Alegre, Brazil; ^4^Sydney Medical School, University of Sydney, Sydney, NSW, Australia; ^5^Institute for Interdisciplinary Innovation in Healthcare, Université libre de Bruxelles, Brussels, Belgium; ^6^Lee Kong Chian School of Medicine, Nanyang Technological University, Singapore, Singapore

**Keywords:** information quality, google, Internet, news, news media, vaccines, autism, public understanding of science

## Abstract

The 1998 *Lancet* paper by Wakefield et al., despite subsequent retraction and evidence indicating no causal link between vaccinations and autism, triggered significant parental concern. The aim of this study was to analyze the online information available on this topic. Using localized versions of Google, we searched “autism vaccine” in English, French, Italian, Portuguese, Mandarin, and Arabic and analyzed 200 websites for each search engine result page (SERP). A common feature was the newsworthiness of the topic, with news outlets representing 25–50% of the SERP, followed by unaffiliated websites (blogs, social media) that represented 27–41% and included most of the vaccine-negative websites. Between 12 and 24% of websites had a negative stance on vaccines, while most websites were pro-vaccine (43–70%). However, their ranking by Google varied. While in Google.com, the first vaccine-negative website was the 43rd in the SERP, there was one vaccine-negative webpage in the top 10 websites in both the British and Australian localized versions and in French and two in Italian, Portuguese, and Mandarin, suggesting that the information quality algorithm used by Google may work better in English. Many webpages mentioned celebrities in the context of the link between vaccines and autism, with Donald Trump most frequently. Few websites (1–5%) promoted complementary and alternative medicine (CAM) but 50–100% of these were also vaccine-negative suggesting that CAM users are more exposed to vaccine-negative information. This analysis highlights the need for monitoring the web for information impacting on vaccine uptake.

## Introduction

Acceptance and uptake of vaccination is important for reaching public health targets. The information available, either from books, television news, newspaper articles, or online sources, has a major impact on how the public perceives vaccines. In this respect, the most impactful information was the publication by Andrew Wakefield in the medical journal The Lancet in 1998, supporting a link between the mumps, measles, and rubella (MMR) vaccine and autism (1). The journal eventually retracted the paper in 2010 (2), because its findings were discredited (3), but its message has become commonplace and remains a significant concern among parents (4).

It has often been pointed out that antivaccine information available on the Internet has a high prevalence and could impact negatively vaccination decisions (5–8). Observational studies have shown an association between exposure to antivaccine information on Twitter (9), and on the Internet in general (10), and a negative perception of vaccine risks. A Canadian study on 250 mothers also reported that reliance on governmental websites, which promote vaccination, is associated with higher vaccination rates (11). It is difficult, however, to draw a causal link from these associations and quantify the impact of online information on vaccine uptake.

Furthermore, the information on the prevalence of antivaccine websites is not consistent. A study in the USA analyzing 89 websites on human papilloma virus (HPV) returned by Google, Yahoo, and Bing reported less than 10% of websites with negative tone about vaccines (12) while one on MMR, also in the USA, reported that searching Google in 2014 returned a proportion of 41% of antivaccine websites (13).

The purpose of this study is to analyze the information available to the public, 20 years on from the publication of the above mentioned Lancet paper, on the link between vaccines and autism. The study does not analyze the impact of online information of vaccination rates or on public health views on vaccines but provides an approach to monitor vaccine-related information on the web. Using a methodology used previously for similar studies, we obtained a sample of the existing information using Google as the search engine (14–17). This captures most information as news outlets, television, books, professional or government organizations, scientific journals, and personal websites or blogs are all online. We sampled the first 200 results returned by Google searching for “autism vaccines,” and analyzed them for the vaccines mentioned, their stance on vaccination, and the source of the website. We also used a standard indicator of health information quality, the JAMA score, to assess their basic trustworthiness index. The JAMA score considers whether a website declares author, date of writing, financial ownership, and whether its information is backed up by references (18).

The analysis was performed in different countries on localized versions of the search engine in different languages (google.com, google.co.uk, and google.com.au in English; google.be in French; google.it in Italian; google.com.br in Portuguese; google.com.sg in Mandarin; google.com.sa in Arabic). This research was done by a pre-existing international research collaboration, and that dictated the choice of the languages or localized versions of Google.

We also investigated the visibility, in terms of ranking, given by the search engine to webpages with a negative tone on vaccines. This has been overlooked by most studies, and it is known that users typically spend a short time on each website (19) and seldom go beyond the first ones in the search engine result page (SERP) (20).

The results indicate differences in the composition of the antivaccine websites across the world and the footprint left by Wakefield’s Lancet paper. They also show differences in the ranking of antivaccine websites in the different localized versions of Google.

## Materials and Methods

We searched the two keywords “vaccines” and “autism” in Google between June and September 2017. It was decided to use only those keywords because we wanted to obtain a sample of the websites returned independently of the expression used. For this reason, we decided not to use questions such as “do vaccines cause autism?” because the results would be different depending on how the question was formulated and we needed to be consistent across the different languages. Although “vaccines” could be synonymous to “immunization,” particularly in the scientific literature, we decided to use the search term “vaccines” as this best represents what the lay public would search on the Internet.

Before performing the search, the investigators deleted cookies and browsing history from their browsers to avoid the results of the search being influenced by previous searches done on the same computer (21–23), although it must be noted that the search engine will still identify the locations where the searches was made from the IP address, and this may customize results. Locations where the searches were performed were as follows: google.com (English), google.co.uk (English), google.it (Italian), and google.com.sa (Arabic), Brighton, UK; google.com.au (English), Sydney, NSW, Australia; google.be (French), Brussels, Belgium; google.com.sg (Mandarin), Singapore; google.com.br (Portuguese), Porto Alegre, Brazil.

The first 200 websites returned in each SERP were transferred to a spreadsheet and then the websites visited individually. When searching google.be, the French terms (vaccins, autisme) were used and any webpage in Flemish would be excluded from the analysis. Webpages that were deemed not relevant, for instance, not mentioning vaccines or aggregators, like those no longer accessible, behind a paywall or requiring registration were excluded from the analysis.

The total number of webpages considered for the analysis were as follows: English (Google.com), 175; English, UK, 188; English, Australia,194; French, 154; Portuguese, 132; Italian, 191; Mandarin, 179; Arabic, 146.

For each website, we recorded the typology of the website using the classification previously described (16, 17). The typologies considered were: Commercial (C), Government (G), Health portal (HP), News (N), No-profit (NP), Professional (P), scientific journals (SJ), as shown in Table 1. Those not fitting any of these categories or difficult to classify are listed as “others” (O). These included blogs, personal websites, or websites not affiliated with any of the other typologies.

**Table 1:** Definitions and examples of typology of websites.

Typology	Description	Examples
Government (G)	Website of a governmental body	nhs.uk, cdc.gov, who.int
Health Portal (HP)	Website that contains information on a variety of health topics	Kidshealth.com, webmd.com
News (N)	A website from newspapers, magazines, or TV	Pbs.org, newsweek.com, arstechnica.com
Non-Profit (NP)	Website from a no-profit organization^a^	Autismcenter.org, avoiceforchoice.org
Professional (P)	Websites created by a health professional organization (medical school, clinic/hospitals, medical board)	Ama.com.au, livewellpediatrics.com
Commercial (C)	Selling of producing drugs, supplements, or other	mercola.com, bodyecology.com
Scientific journal	Academic journals	Sciencedirect.com, nature.com

^a^In the UK, they indicate a “registered charity” number, in the USA “tax-deductible 501(c)(3) organization.”

To assess the JAMA score, we searched the webpage for the presence of the following information: author, date, references, owner of website (18).

We also annotated webpages according to the following features:

(1) The name of the vaccine mentioned; (2) the overall stance on vaccines (positive, negative, or neutral); (3) the chemicals or adjuvants mentioned; (4) whether the page mentioned complementary and alternative medicine (CAM) and its stance toward it (positive, neutral, or negative); (5) whether religion was mentioned; (6) whether the page contained a testimonial (e.g., a personal story); (7) whether a celebrity was mentioned. For websites associated with the typology “News,” we recorded the most mentioned stories in each SERP.

### Statistical Analysis

When indicated, statistical analysis was performed using GraphPad Prism version 7 for Windows (GraphPad Software Inc., La Jolla, CA, USA).

A two-tailed Fishers Exact test was used when comparing frequencies; when comparing multiple groups, the Bonferroni correction for multiplicity was applied.

When comparing JAMA scores across more than two groups, ANOVA was performed followed by Kruskal–Wallis test corrected for multiplicity by controlling the false discovery rate using the method of Benjamini and Hochberg.

A Pearson correlation coefficient test was used to assess the correlation between two variables, following D’Agostino and Pearson normality test (when the number of samples was too small, a Kolmogorov–Smirnov test was used to determine normality, a pre-requisite for the Pearson’s test). For non-normally distributed samples, correlation was assessed using a Spearman Rank test. An alpha value of 0.05 was used for all statistical tests unless otherwise specified.

The statistical test used is described in the text or in the legends to figures and tables.

Word count to detect the number of occurrences of the names of celebrities was performed using natural language processing. Briefly, text corpora were extracted using WebBootCaT, an online tool for bootstrapping text corpora from Internet. Then word counts were obtained using the corpus analysis software Sketch Engine by Lexical Computing, Brno-Královo Pole, Czechia (24).

The raw data containing the list of websites analyzed and how they were annotated in provided in Data Sheet S1 in Supplementary Material.

## Results

### Focus on MMR

Because we only used the word “vaccine” without specifying further, we first analyzed the vaccines mentioned in the webpages returned. As shown in Table 2, MMR was the most discussed vaccine, as expected, followed by influenza, viral hepatitis, diphtheria–tetanus–pertussis (DTP), poliomyelitis, Haemophilus influenza b and meningococci, HPV. However, there were differences between the various languages. The largest spread of vaccines mentioned was observed in Mandarin, while webpages in Arabic only mentioned MMR and influenza. Mandarin webpages also mentioned BCG while those in Portuguese mentioned Yellow fever and measles.

**Table 2:** Vaccines discussed by webpages in the different search engine result page (SERPs).

	Com	UK	AUS	FR	IT	Man	Port	ARA	Total
Mumps, measles, and rubella	123	133	112	96	93	116	88	71	832
Influenza	23	20	3	11	6	21	9	4	100
Hep	16	10	10	13	2	34	13	0	98
Diphtheria–tetanus–pertussis	10	9	4	4	0	37	8	0	72
Polio	5	10	5	6	18	25	0	0	69
Hib/Men	8	4	3	2	4	29	0	0	50
Human papilloma virus	6	6	0	1	2	8	0	0	23
Chickenpox	4	3	2	5	2	9	0	0	25
Pertussis	10	5	2	7	0	0	0	0	24
Rotavirus	3	2	0	1	2	10	0	0	18
Pneumococcal	3	3	0	5	0	10	0	0	21
Smallpox	4	2	0	20	4	4	0	0	34
BCG	0	0	0	0	0	10	0	0	10
Yellow fever	0	0	0	0	0	0	3	0	3
Measles	0	0	0	3	0	0	3	0	6

*Values indicate the number of webpages in each SERP mentioning a specific vaccine. Color intensity indicate the frequency vaccines are mentioned in each SERP*.

### Typologies of Websites

Table 3 shows the composition of the SERP in terms of website typologies. In all SERPs, most websites (60–80%) were “news” or “other” (including non-affiliated websites, blogs etc.). Websites from governmental (e.g., national and international public health services, health ministries, CDC, FDA, etc.) or inter-governmental organizations (e.g., WHO) were not highly represented, their frequency ranging from 1.3% (French) to 6.7% (English/Australia).

**Table 3:** Composition of the search engine result page (SERP) by typology of webpages.

Typology	Google.com	UK	AUS	FR	IT	Man	Port	ARA
Comm	4.0	5.3	3.1	6.3	0.0	2.2	4.5	0.0
Gov	1.7	6.4	6.7	1.3	3.1	5.0	3.0	3.4
HP	3.4	3.7	6.2	1.9	4.2	10.1	10.6	11.6
News	41.7	30.3	26.3	31.6	49.7	36.9	31.1	34.9
NP	11.4	13.8	10.8	7.0	6.3	7.3	2.3	2.1
Other	26.9	26.6	29.9	41.1	32.5	31.8	29.5	39.0
Prof	7.4	6.9	10.3	8.9	4.2	5.6	17.4	8.2
ScJ	3.4	6.9	6.7	1.9	0.0	1.1	1.5	0.7
Total	100.0	100.0	100.0	100.0	100.0	100.0	100.0	100.0

*Data are expressed as percentage of the total for each SERP. Color intensity indicate the frequency of the different typologies in each SERP*.

Non-profit organizations, health portals and professional websites followed in various proportion. Commercial websites had a presence (except in Italian) between 2 and 6%. SJs online were present in a significant percentage (3–7%) only in the three SERPs in English, which is not surprising if we consider that scientific literature is mostly in English.

On the other hand, the pattern in the top 10 websites is completely different (Table 4). Commercial websites are not present in the top 10 websites returned by Google. In many SERPs the frequency of government websites was 10–30%, higher than that in the whole search. News websites, representing 30–40% of the SERPs in English, were also less frequent (0–10%) in the top 10. The exception was the SERP in French where news websites represented 60% of the top 10 websites compared to 31% in the whole SERP, and a similar trend was observed in Portuguese (40% in the top 10, 30% in the whole search).

**Table 4:** Composition of the top 10 webpages by typology.

Typology	Google.com	UK	AUS	FR	IT	Man	Port	ARA
Comm	0	0	0	0	0	0	0	0
Gov	1	2	3	0	0	1	0	1
HP	1	0	1	0	1	2	1	1
News	1	1	0	6	3	2	4	2
NP	3	4	1	1	1	0	1	1
Other	1	1	1	3	3	4	2	5
Prof	2	0	3	0	2	0	2	0
ScJ	1	2	1	0	0	1	0	0

*Data indicate the number of websites (total = 10). Color intensity indicate the frequency of the different typologies in each SERP*.

### Testimonials, Celebrities, and CAM

We investigated whether websites contained a testimonial (personal story), mentioned a celebrity, or mentioned CAM.

**Figure 1:**
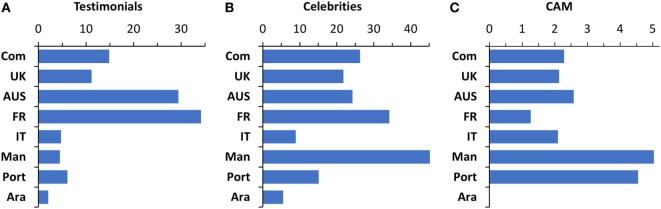
Percentage of webpages containing testimonials **(A)** or mentioning celebrities **(B)** or complementary and alternative medicine **(C)**.

As shown in Figure 1A, testimonials were present in around 30% of websites returned by the Australian and French Google searches, but were much less frequent in Italian, Mandarin, Portuguese, and Arabic websites.

Celebrities (Figure 1B) were present with high frequency in English, French, and Mandarin websites. The celebrities most frequently mentioned, and present in most languages, were Donald Trump (present in all SERPs, ranging from 27 webpages in Australia, 19 in UK, 18 in Mandarin, with a minimum of 1 in Arabic), Jenny McCarthy (present in SERPs in English, Portuguese, and Mandarin, 19 times in Australia, 12 in google.com, and 9 in UK), Robert F. Kennedy Jr. (23 webpages in Australia, 17 in google.com, 16 in UK), and Robert De Niro (present in all searches except Mandarin and Portuguese). Other celebrities mentioned were Dan Burton, Jim Carrey, Chuck Norris, and Luc Montagnier. Other names were language- or country-specific. In French, Martine Ferguson-André was mentioned in 23 websites while Agnès Buzyn was mentioned by 7 webpages. In Italian, Beatrice Lorenzin, was mentioned in 19 webpages. A short description of the main celebrities mentioned is given in Table 5. Interestingly, most of them were named by vaccine-positive or -neutral websites when describing the antivaccine movement.

**Table 5:** Celebrities most mentioned in the search engine result pages.

Name	Context
Donald Trump	US president, suggest vaccine cause autism on Twitter

Robert F. Kennedy Jr.	US Environmental attorney, claim links between vaccines and autism, rumored to be appointed by Donald Trump to lead a committee on vaccine safety

Jenny McCarthy	US actress and Playboy model, blames vaccination for his son’s autism

Robert De Niro	US actor, founder of Tribeca festival. He has a son with autism and was linked to belief of the link between vaccines and autism and critical of the Center for Disease Control. He reversed his initial decision to include the film “Vaxxed” from the festival

Jim Carrey	US actor with autistic son (from Jenny McCarthy), led a “green our vaccines” march in Washington, DC and is critical of the Center for Disease Control

Chuck Norris	US actor, accused government to hide data on links between vaccines and autism

Dan Burton	US representative, grandfather of a child with autism, believer that thimerosal causes autism. Previously expressed support of laetrile, a complementary therapy for cancer

Luc Montagnier	French scientist, Nobel prize for the discovery of HIV. Attended vaccine skeptical conferences and highlighted an association between vaccine and autism (however, he warned that this may not mean causation). Previously linked to condescendence toward homeopathy

Martine Ferguson-André	French politician. Suspects vaccines caused his son’s autism

Agnès Buzyn	French health minister, introduced 11 vaccines compulsory

Beatrice Lorenzin	Italian health minister, passed a law making 10 vaccines compulsory

Few websites mentioned CAM, and their frequency was higher in Mandarin and Portuguese websites (4–5%), while in other SERPs, they accounted for no more than 2% of the websites (Figure 1C).

### Stance on Vaccines

The most important aspect of the content analysis was to assess the stance of websites toward vaccines, whether pro-vaccine, vaccine-negative, or neutral. A pro-vaccine stance would be that of websites promoting vaccination or denying the causal link with autism. A vaccine-negative stance would be that of supporting a link with autism or discouraging vaccinations, like the so-called “anti-vaxxers.” An example of neutral stance would be that of a news website reporting the existence of this controversy or a scientific paper reporting findings from an epidemiological study.

**Figure 2:**
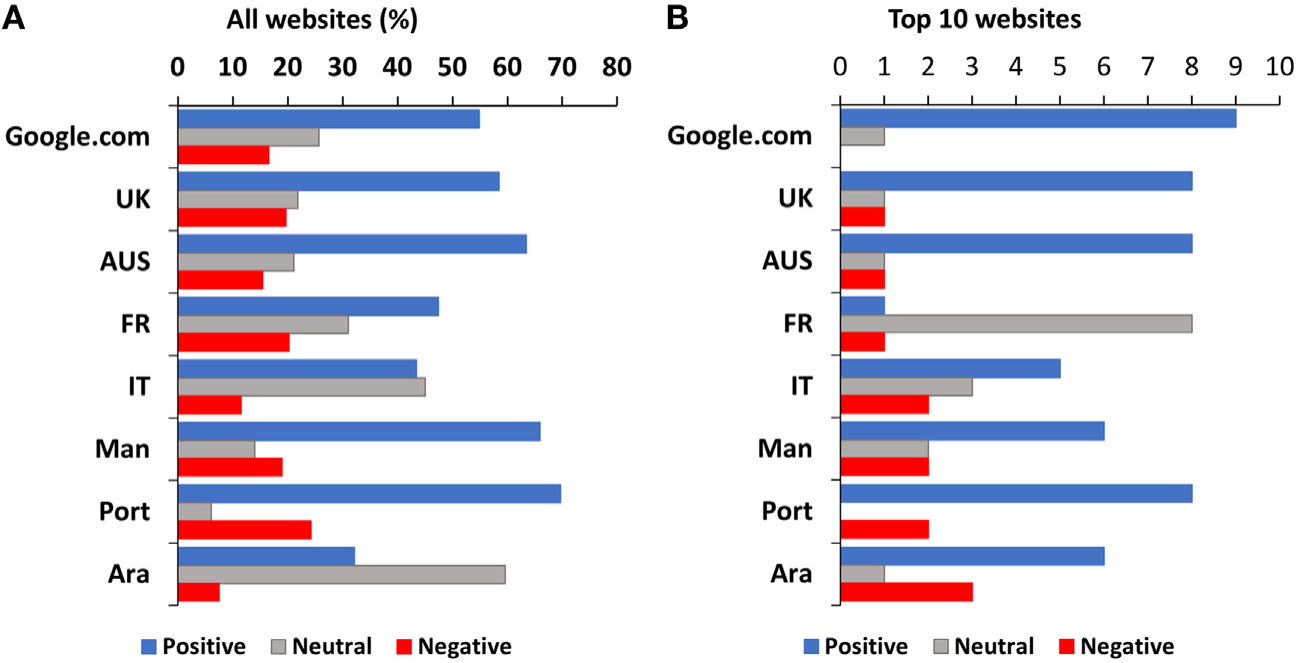
Webpages with different stance on vaccines in the entire search engine result page **(A)** and in the top 10 webpages **(B)** returned by Google. Data are expressed as percentage of websites for the entire search or number of websites in the top 10.

Figure 2 reports the presence of total websites that are pro-vaccine, neutral, or vaccine-negative in the whole SERP (panel A) and in the top 10 websites returned by Google (panel B). The frequency of vaccine-negative webpages in the top 10 results was lower than that observed in the rest of the SERP in most languages except for Italian (11% in the whole SERP, 20% in the top 10) and Arabic (7.5% in the whole SERP, 30% in the top 10, *P* = 0.0485 by Fisher’s test). The frequency of pro-vaccine websites in the top 10 was significantly higher than in the rest of the SERP in google.com but lower in google.be; Fisher’s test, *P* = 0.0472 and 0.0220, respectively.

**Figure 3:**
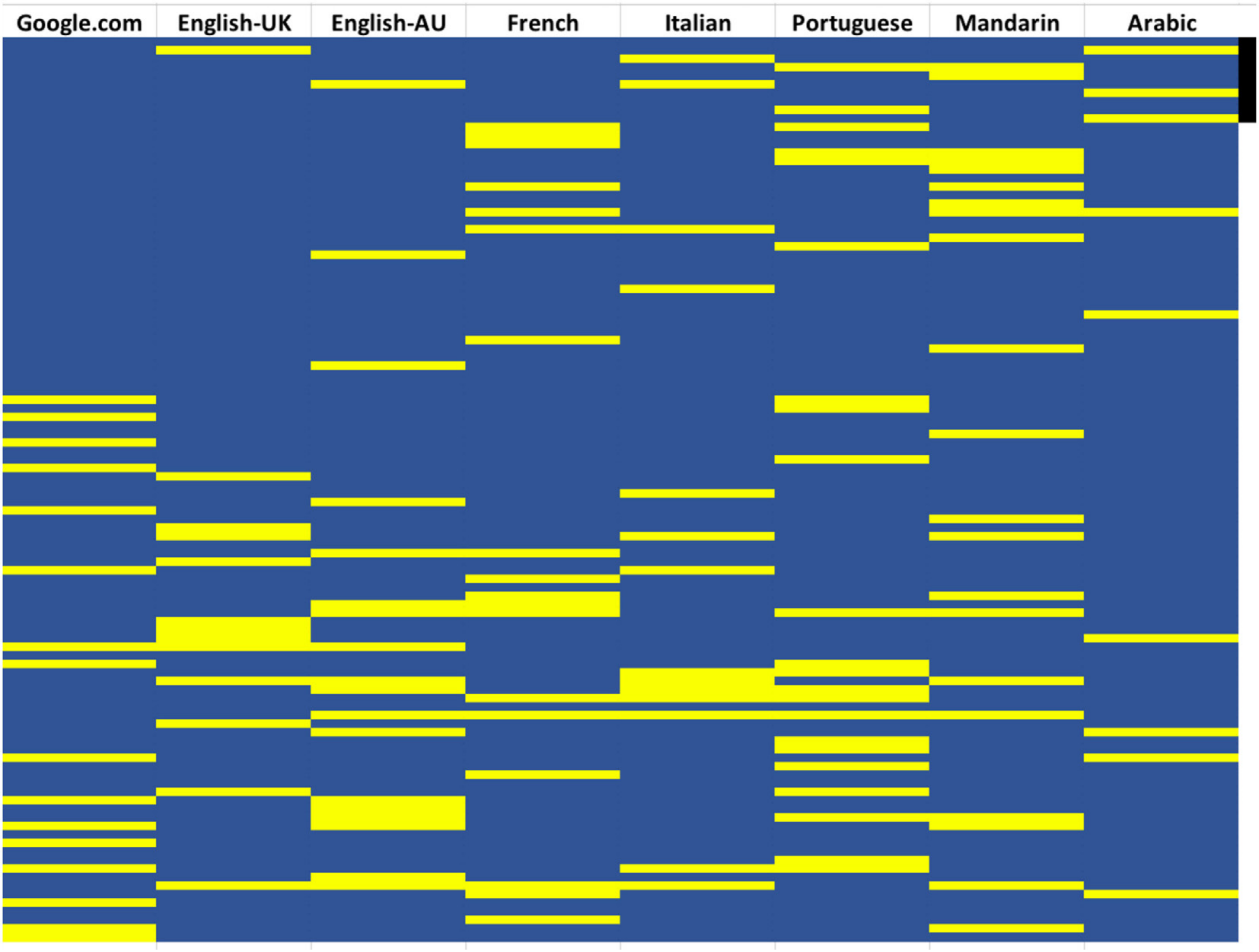
Visualization of the ranking of webpages with a negative stance on vaccines in the first 100 websites in each search engine result page (SERP). Webpages are listed in the same order they are ranked in the SERP. Yellow, vaccine-negative websites; blue vaccine-positive or -neutral. The black bar on the right indicate the top 10 webpages.

Figure 3 provides a visual representation of the ranking of the vaccine-negative websites (in yellow) in the first 100 websites across the different SERPs. There is a clear trend for searches in English websites which give a lower visibility to vaccine-negative webpages.

The observed frequency of vaccine-negative webpages across the different typologies of websites is reported in Table 6. For each language SERP, we color-coded values based on how the observed frequency of vaccine-negative URLs in that typology compared with the expected frequency (the overall percentage of vaccine-negative websites in the whole SERP). In almost all SERPs, a higher proportion than expected of commercial websites were vaccine-negative in stance (up to 71.4% were observed in google.com compared with 16.6% expected). It should be noted, however, that commercial websites account for only 2–6% of the total websites returned, and they never appear in the top 10, as shown in Tables 3 and 4. A higher frequency of websites classified as “other” were observed to be vaccine-negative in their stance (up to 40% of websites in the UK). This is particularly relevant as this website typology accounts for about one-third of the total SERPs. As expected, there were no vaccine-negative websites among the government typology, and very few in the professional typology (average of all SERPs, 6.9%). Vaccine-negative views were also infrequent in news websites (averaging 5.2% all SERPs).

**Table 6:** Frequency of vaccine-negative webpages in each typology.

	google.com	UK	AUS	FR	IT	Man	Port	Ara
Comm	71.4	60.0	16.7	77.8	0.0	25.0	16.7	0.0
Gov	0.0	0.0	0.0	0.0	0.0	0.0	0.0	0.0
HP	0.0	0.0	16.7	0.0	12.5	27.8	7.1	0.0
News	6.8	7.0	2.0	0.0	2.1	12.1	7.3	3.9
NP	10.0	11.5	4.8	30.0	0.0	15.4	33.3	0.0
Other	34.0	40.0	39.7	30.8	30.6	29.8	56.4	15.8
Prof	7.7	7.7	5.0	7.1	0.0	10.0	17.4	0.0
ScJ	0.0	23.1	7.7	0.0	0.0	0.0	0.0	0.0

**Average vaccine-negative in the total search engine result page (SERP)**								
	16.6	19.7	15.5	19.6	11.5	19.0	24.2	7.5

*Data indicate the percentage of vaccine-negative webpages in each typology. Cells are color coded to show difference from “expected” based on the frequency in the total SERP shown in the bottom row (red, above the expected frequency; green, below the expected frequency)*.

**Figure 4:**
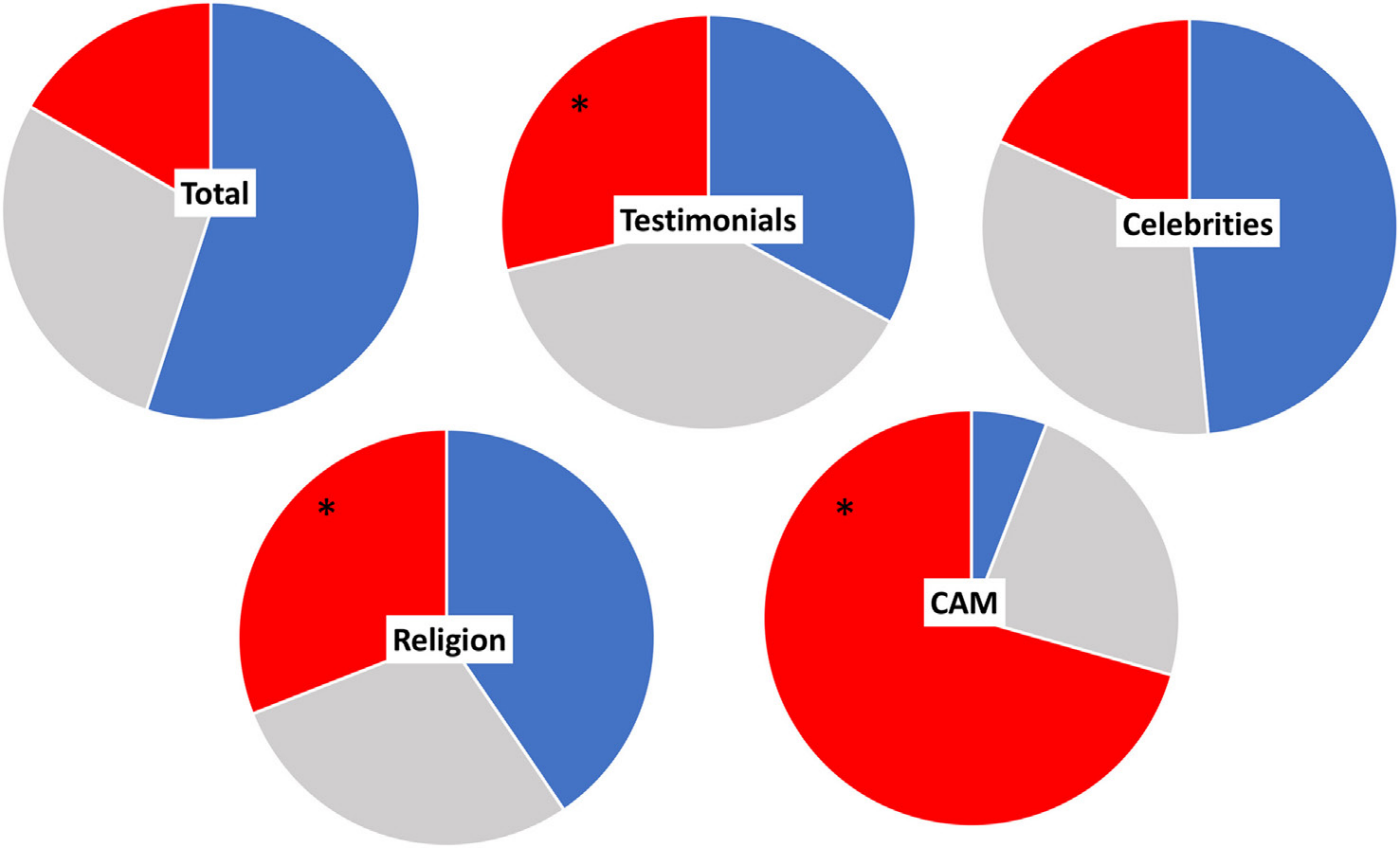
Vaccine stance in webpages from all search engine result page (SERPs) mentioning testimonials, celebrities, religion, or complementary and alternative medicine. Blue, vaccine-positive, gray, neutral, red, negative. * Denotes a higher frequency of vaccine-negative webpages compared to the total SERP (*P* < 0.05 by Fisher’s test).

We also analyzed whether the mention of testimonials, celebrities, CAM, or religion was associated with a particular stance on vaccines. Figure 4 represents the stance on vaccines in all webpages from all SERPs mentioning testimonials, celebrities, CAM, or religion. The frequency of vaccine-negative websites was significantly higher in webpages reporting testimonials (*P* = 0.0002 by Fisher’s test), CAM (*P* = 0.0001), or religion (*P* = 0.02) when compared to the total. On the other hand, websites mentioning celebrities had a similar pattern as the total search, indicating that even celebrities such as Trump were not mentioned in a vaccine-negative context.

### Adjuvants

There is a diffuse concern that the chemicals, including adjuvants and preservatives, added to vaccines to act as adjuvants may be a cause of autism. We, therefore, took note of when a webpage mentioned the presence of it in the text. The chemical name occurring with the highest frequency was thimerosal (441 webpages, 60% of total), followed by the partially synonym mercury (184 webpages, 25% of total), aluminum (101, 14%), and formaldehyde (15, 2%). These adjuvants and preservatives were mentioned in a large proportion of the websites: 56% in Google.com, 50% in UK, 93% in Australia, 58% in French, 28% in Italian, 45% in Mandarin, 71% in Portuguese, and 32% in Arabic.

A sub-analysis of the adjuvant mentioned by websites and the stance of the website on vaccines showed that vaccine-negative websites mentioned aluminum with a frequency that was nine-times higher than pro-vaccine, and four-times higher than neutral, websites (Table 7). Although this trend was also observed for “mercury,” it was not observed for “thimerosal.”

**Table 7:** Main chemicals mentioned in webpages with different stance.

	Positive	Neutral	Negative
Thimerosal	274 (37%)	86 (22%)^a^	81 (36%)
Mercury	92 (12%)	32 (8%)	60 (27%)^a^
Aluminum	21 (3%)^a^	23 (6%)	57 (25%)^a^
Formaldehyde	7 (1%)	2 (1%)	6 (3%)

Total	744 (100%)	384 (100%)	225 (100%)

*Number of webpages mentioning a chemical of all the webpages with that stance on vaccines. Percentage is given in parenthesis*.

*^a^Significantly different frequency compared to that of the other two groups combined (*P* < 0.05 by Fisher’s test with Bonferroni correction for multiplicity for 12 comparisons)*.

### News

Because of the high frequency of news websites, accounting for about one-third of all SERPs, we have summarized in Table 8 the main topics covered by these websites.

**Table 8:** Main topics in news webpages.

Search engine result page	Topic	Examples
Google.com	Tribeca film festival and the anti-vaccine film “Vaxxed”	https://www.nytimes.com/2016/03/26/health/vaccines-autism-robert-de-niro-tribeca-film-festival-andrew-wakefield-vaxxed.html https://www.statnews.com/2016/03/31/vaxxed-vaccine-autism-movie/(Archived at: http://www.webcitation.org/6ww1JScrv)

	Donald Trump and political debate on vaccinations	https://www.usnews.com/news/articles/2017-01-24/donald-trumps-health-care-pick-rejects-claims-that-vaccines-cause-autism (Archived at: https://web.archive.org/web/20180202164318/https://www.usnews.com/news/articles/2017-01-24/donald-trumps-health-care-pick-rejects-claims-that-vaccines-cause-autism) http://uk.businessinsider.com/trump-vaccines-autism-wrong-2017-1?r=US&IR=T (Archived at: http://www.webcitation.org/6ww0yJnpi)

	Theory that the Center for Disease Prevention and Control (CDC) have withheld evidence that that African-American boys are at an increased risk of developing autism	https://www.colorlines.com/articles/new-documentary-alleges-cdc-withheld-proof-link-between-vaccines-and-autism-black-boys (Archived at http://www.webcitation.org/6ww0fb2AC)

Portuguese	Report the story of the origin of the myth of the link autism-mumps, measles, and rubella (MMR) Wakefield paper	https://web.archive.org/web/20180202161923/https://g1.globo.com/bemestar/noticia/a-historia-que-deu-origem-ao-mito-da-ligacao-entre-vacinas-e-autismo.ghtml https://web.archive.org/web/20180202162138/http://www.bbc.com/portuguese/geral-40663622

French	Report on a new law to make 11 vaccines compulsory in France, and of the opposition by Martine Ferguson-André, member of Europe Ecologie-les Verts	http://rmc.bfmtv.com/emission/vaccins-obligatoires-le-lien-entre-le-vaccin-contre-la-rougeole-et-l-autisme-ne-tient-pas-scientifiquement-1247033.html [Archived at: http://www.webcitation.org/6tSAGAQaw] http://www.la-croix.com/Sciences-et-ethique/Sante/Vaccination-pourquoi-parents-denfants-autistes-souhaitent-poursuivre-laboratoires-2017-07-24-1200865117 [Archived at: http://www.webcitation.org/6tUT5QS7t]

Italian	Reports of courts cases and final sentences of the Supreme Court in June 2016 and July 2017, which denied the causal link between vaccines and autism. Most news take the stance that connection between vaccines and autism is a hoax (“bufala”) except one vaccine-negative article in “Corriere Quotidiano”	https://www.agi.it/salute/vaccini_bambini_e_autismo_storia_di_una_bufala-1987339/news/2017-07-26/(archived at http://www.webcitation.org/6tofW4vYP) http://www.repubblica.it/salute/2017/07/25/news/cassazione_non_c_e_correlazione_tra_vaccini_e_autismo_no_al_risarcimento_-171599464/(archived at http://www.webcitation.org/6wAj2KM3O) http://www.corrierequotidiano.it/1.67940/salute-e-medicina/toscana-siena/3715/vaccini-e-autismo-cassazione-nega-corte-europea-avvalla (archived at http://www.webcitation.org/6wApTvo0b)

	Report on the law, approved by the Italian Parliament in July 2017, making ten vaccinations compulsory for all children aged 10–16	http://www.metronews.it/17/09/07/dietrofront-del-veneto-stop-alla-moratoria-vaccini.html (archived at: http://www.webcitation.org/6xTn4tELD)

Mandarin	China Shandong Illegal Vaccine Scandal on vaccines purchased from illegal sources and not stored properly	http://www.zaobao.com.sg/wencui/politic/story20160324-596554 (archived at: http://www.webcitation.org/6xTSPfm4k)

	Donald Trump’s stance on vaccines	http://hssszn.com/archives/17810 (Archived at http://www.webcitation.org/6tUAlNx7P) http://3g.forbeschina.com/review/201204/0016345.shtml (Archived at: http://www.webcitation.org/6tU9cS9qm)

	Andrew Wakefield. Talks about the revocation of his medical license and his fraudulent research paper published in The Lancet linking MMR vaccines to autism, which has since been withdrawn	http://www.webcitation.org/6tUNCaBbf http://www.webcitation.org/6tUMuQHdk https://read01.com/LNedyP.html (Archived at: http://www.webcitation.org/6tUPLRZEM) http://m.6park.com/index.php?act=wapnewsContent&nid=239399 (Archived at: http://www.webcitation.org/6tUNliETc)

As mentioned above, vaccine-negative news webpages were less frequent than expected in the whole SERPs. Vaccine-negative news articles were highest in Mandarin, Portuguese, UK, and google.com (12.1, 7.3, 7, and 6.8%, respectively) and lowest in French, Australian, Italian, and Arabic (0, 2, 2.1, 3.9%, respectively) webpages.

### JAMA Score

**Figure 5:**
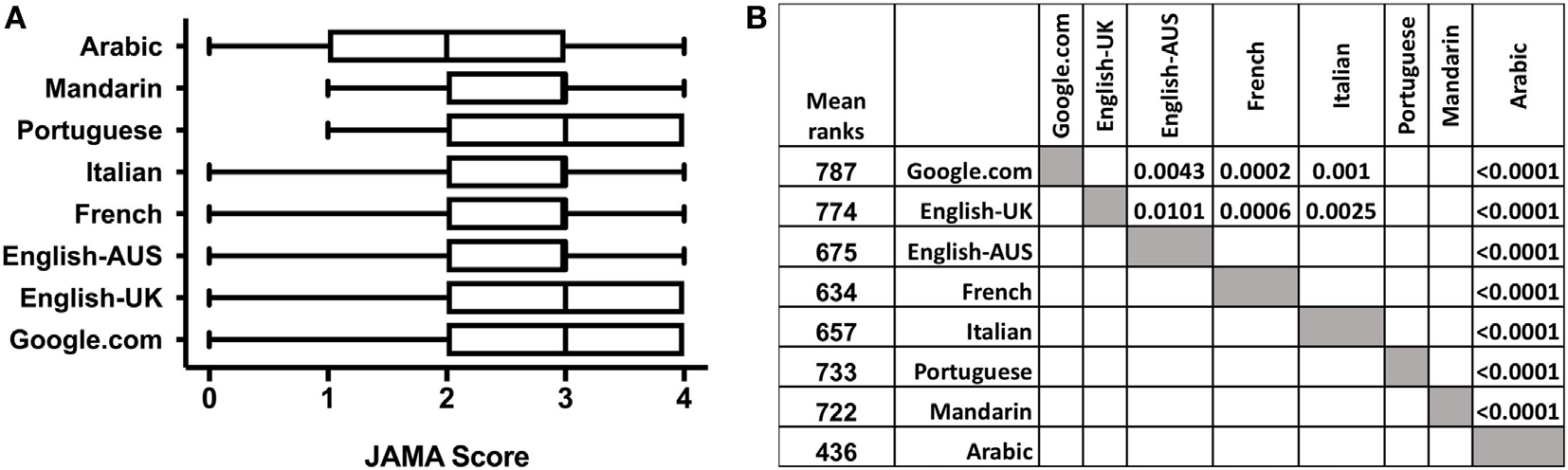
JAMA score of webpages in the different search engine result pages (SERPs). **(A)** Box-and-whiskers graph indicate median, 25 and 75% percentiles, minimum and maximum. **(B)** Multiple comparison of different SERPs. *P*-values are reported only for statistically significant differences. Multiple comparison of JAMA scores among the different SERPs was performed using ANOVA followed by Kruskal–Wallis test corrected for multiplicity by controlling the false discovery rate using the method of Benjamini and Hochberg for 28 comparisons.

The median JAMA score for all SERPs is shown in Figure 5. The Arabic SERP had a significantly lower JAMA score than any other SERP. Google.com and Google.co.uk had a significantly higher JAMA score than the SERPs in English-Australia, French, and Italian.

We also analyzed, for each SERP, the JAMA score of vaccine-positive, -neutral, or -negative and could not find any difference in the JAMA score of websites with different stance on vaccines (data not shown). Furthermore, for any SERP, we could not find any significant difference in the JAMA score of the top 10 websites compared to the rest of the SERP.

## Discussion

The varied composition of the SERP returned by Google, with only 30% being non-affiliated websites or blogs, and the rest representing a wide range of news outlets, professional or government organizations, and scientific journals, represents a good sample of the information on the topic of vaccines and autism that the public is exposed to.

Because we analyzed the first 200 websites returned by Google, the list is not just a sample of all that is available in what has been called the infosphere (25), but it also reflects the visibility, or ranking, given by Google. For this reason, we did not just look at the composition of the SERP but also how webpages are ranked, particularly, the first 10 results that are more likely to be read (26).

Despite retraction of his paper in 2010, Dr. Wakefield is still highly mentioned (a word count found his name recurring 462 times in the Google.com search, 551 in UK, 706 in Australia, 378 in French, 361 in Italian, 21 in Arabic, 195 in Portuguese, and 11 in Mandarin). Although his original paper did not appear in any SERP, a letter he published in the Lancet in 1999 was present in both the UK and the Australian SERP (but not Google.com). In French, two websites (one Belgian and one French) displayed a video of Andrew Wakefield’s interview with subtitles in French (http://initiativecitoyenne.be/2017/02/vaccins-autisme-le-dr-andrew-wakefield-repond-aux-accusations-et-aux-calomnies.html, accessed 19/03/2018 and archived at https://web.archive.org/web/20180319102307/http://initiativecitoyenne.be/2017/02/vaccins-autisme-le-dr-andrew-wakefield-repond-aux-accusations-et-aux-calomnies.html; http://www.agoravox.tv/tribune-liber/article/vaccination-et-autisme-dr-andrew-72269, accessed 19/03/2018 and archived at https://web.archive.org/web/20180319102342/http://www.agoravox.tv/tribune-liber/article/vaccination-et-autisme-dr-andrew-72269.).

It is important to be aware that the autism-MMR scare was not borne out of an obscure sect but from scientific papers published in respectable and authoritative journals, leading to a widespread concern even among health professionals.

This seems to be true today when articles published in academic journals of varied respectability can have a significant impact as they may be perceived as providing a scientific basis for antivaccine, or just vaccine-skeptical, positions. A study has shown that, in the US, a drop in the MMR vaccination rate was observed soon after the publication of original scientific reports, even before this was the subject of media coverage (27). These may also be ranked higher by search engines because scientific articles may be considered authoritative and, therefore, proxies for high quality information.

It may be surprising that in the UK and Australian websites, but not in Google.com, a proportion of SJs were vaccine-negative. As mentioned above, very few websites of SJs were present in non-English SERPs, not surprisingly as scientific articles are usually in English. Of the six scientific articles in Google.com, none were vaccine-negative, whereas UK and Australian websites (13 scientific articles each) had some vaccine-negative scientific articles (three and one, respectively). In the UK SERP, three vaccine-negative scientific papers were found. One was a 2002 paper in *LabMedicine*, published by the Oxford University Press and the American Society for Clinical Pathology and, to our knowledge, never retracted (28); a second a letter by Wakefield published in the Lancet in 1999 in response to criticism over his previous paper (29); a third is a 2017 editorial published in the “*Madridge Journal of Vaccines*,” a journal published in the US but, unlike *The Lancet* and *LabMedicine*, not listed by PubMed and the National Library of Medicine (30).

In particular, the 2002 paper published by Oxford University Press was ranked second in the UK SERP. Repeating the “autism vaccines” search on Google.co.uk 6 months later still returned this article second in the ranking (data not shown). This online article was not found in Google.com or in the Australian SERP.

In the Australian search, two websites were collections of scientific papers supporting a causal link between vaccines and autism, a third the Wakefield letter mentioned above, and a fourth a paper by the organization “Informed Consent Action Network” that, even if not published in a journal, and it might be questionable whether it could be legitimately defined a scientific paper as it is unclear whether it was peer reviewed, has all the features of a scientific review. Classifying these papers as vaccine-negative was a shared but subjective decision of the authors who reviewed those websites, and we provide the references in Data Sheet S1 in Supplementary Material in case the reader wishes to reassess our coding from a different perspective.

As noted in a Nature editorial by Leask (31), “just four months after the publication that triggered the MMR scare, 13% of general practitioners and 27% of practice nurses in north Wales thought it very likely or possible that the vaccine was associated with autism (32)”. Leask noted that, to improve uptake of vaccinations, we should engage “fence-sitting parents” (31). This means that pro-immunization information needs to address those issues and concerns that anti-vaccine websites raise, such as the mention of aluminum or mercury as a component of thimerosal, as highlighted by our study. Furthermore, the present study also advocates the dissemination of pro-vaccine information on the same websites typologies that perpetuate the “fake science” that vaccines cause autism.

Despite the science behind it being discredited, there are several reasons as to why the association between the MMR vaccine and autism is still present amongst the lay public. Flaherty pointed out that this is partly due to autism being a complex condition without a single, established causal mechanism (33). It should be noted that a search of websites mentioning “vaccines and autism” returns websites mentioning other vaccines, not just the MMR, as this could suggest a potential extrapolation of the link with autism to other types of vaccines.

The strong association between vaccine-negative stance and CAM, as well as commercial websites often selling “natural products,” confirms that cultural factors may reinforce an antivaccine stance by the association of vaccines with capitalism, big pharma, and profit.

Another finding of the present study is that government organizations accounted for only 1.3–6.7% of websites (Table 3). This is markedly less than what we found previously in a study on influenza vaccine where governmental websites represented 17% of the SERP in English and 42% of that in Italian (16). The reason for this is probably that, in the present study, we specifically introduced the search term “autism,” which may not be mentioned in most of the government websites unless for educational purpose, which is to explain that there is no link to autism. The other possibility is that, in the case of influenza, there is a strong vaccination campaign because it is done on a voluntary basis, while the MMR is either part of the routine immunization schedule of babies (e.g., UK) or compulsory (e.g., Italy since 2017 or France for babies born after 01/01/2018).

The fact that Trump is the most frequently mentioned celebrity reminds us of the difference between countries, where in some countries antivaccine sentiment is prevalent among alternative, left-wing groups, and right-wing, individualist, groups in others. We could not find a significant association between mention of religious issues and sentiment about vaccines. In fact, religious beliefs may be important in the confidence in vaccines (34), although this may be a confounder as there are few religious groups who officially reject vaccinations (35).

The fact that news outlets represent 30–50% of the websites indicates that the link between vaccine and autism is a topical and newsworthy topic. From this point of view, it is reassuring that news websites returned by Google have a low frequency of vaccine-negative articles. This is not to say that there are no antivaccine news articles (many vaccine-negative articles have been published by top tabloid newspapers in the UK) but rather that these are not given visibility by the algorithm used by Google.

However, the information quality criteria used by Google do not always penalize vaccine-negative websites. This study shows that, while in Google.com the first vaccine-negative webpage came up only as 43rd, in the local UK and Australian SERPs some were found in the first 10 websites, and this was even more marked in non-English SERPs.

Interestingly, this is similar to what we observed in a previous research where we analyzed the information returned by Google on influenza vaccine or influenza prevention in English and Italian. While in google.com in English there were no vaccine-negative websites or websites promoting non-evidence-based medicine approaches to influenza prevention, this was not true for a search in Italian (16).

Of course, here we only use Google as a mesh to collect a sample of the web and the websites returned in the SERP might just reflect “what is out there.” However, it is important to note that the overall frequency of vaccine-negative webpages was not so different in the different SERPs, and we have no explanation for this observation. One wonders whether the vaccine-negative study published in a SJ was ranked high in the UK SERP because the publisher is Oxford University Press, or whether the one in the top 10 in the Australian SERP was ranked high because the .org domain was taken as a proxy of authority and quality. It is also possible that the higher ranking of vaccine-negative webpages in some SERPs is due to the fact that they receive a high number of clicks in that country or language.

Another interesting finding of this study is the difference in the JAMA score of different SERPs. Websites in Arabic showed the lowest JAMA score than all other languages. Websites from Google.com and Google.co.uk ranked higher than those from the localized versions in English-Australia, French, and Italian. The fact that the mean JAMA score of websites returned in the Australian SERP is also significantly lower than that of those returned by Google.com or Google.UK seem to exclude that the language alone explains the difference.

One obvious question is how much the antivaccine information impacts on the uptake of vaccines. Data from the Organization for Economic Co-operation and Development (OECD) show that, in 2015, Italy had the lowest vaccination rate for measles (85%), People’s Republic of China the highest (99%), Australia and France 91%, the USA 92%, the UK 95%, Belgium and Brazil 96%, Portugal and Saudi Arabia, 98% (36, 37). The low immunization rate is the reason why the Italian government made the MMR vaccine compulsory in July 2017, France followed in 2018 and Australia is also going along that route.

We assessed whether there was a correlation between the percentage of vaccine-negative webpages from Figure 2A and either the safety-related skepticism in the countries analyzed (34) or with the uptake of measles vaccination in 2016 (data from https://data.worldbank.org/indicator/SH.IMM.MEAS). There was no statistically significant correlation using the Spearman-Rank test or the Pearson correlation coefficient (data and results of the statistical analysis are provided in Data Sheet S2 in Supplementary Material).

It should also be noted that the search in Mandarin was performed using the localized version of Google in Singapore; because the Google search engine is not available in the People’s Republic of China, our results cannot be extrapolated to the information available in that country. We should also bear in mind that most of the languages investigated are not specific to a single country. Hence, making correlation between webpages in one language and vaccination rate or sentiment in one country, is not immediate.

This lack of correlation might support the view that the impact of online information on vaccination acceptance may be exaggerated. For instance, a study among French mothers reported that the main source of information on vaccination is the family physician or pediatrician (84–90%) and the Internet accounts for only 10–12% (8), while a study on 1737 Canadian parents showed that, to obtain trustworthy and reliable information on vaccines, 68% of them would ask a physician, and just 27% the Internet (38). If we also consider the fact that only a small percentage of parents refuse to vaccinate their children, one could conclude that we should not overestimate the impact of webpages with a vaccine-negative stance. Other issues may be at the basis of vaccine skepticism such as the perceived role of big pharma and governments or the underestimation of potential risks, as in the case of the dengue vaccine (39).

A major limitation of this study is that we only looked at webpages and did not investigate social networks. Studies have previously explored this area of the Internet and have analyzed their features in English and French (6, 8). Another limitation of the present study is that we analyzed the sample of the online information on the topic but not all websites will have the same impact. Even within the first ten results, readers may just briefly glance through them using clues to decide what to read. To assess which top-ranking websites attract attention of the user and are actually read, research should be undertaken by asking volunteers to rank websites or, alternatively, their attention could be monitored using eye-tracking software (40). A further limitation of our study is that we used the same, neutral, search string (“vaccine autism”) without taking into account potential differences in the most searched terms used, which could well be different in different languages. It is likely that users could find more biased information by using more negative search terms, although a recent study using eye-tracking software to investigate the search behavior of 56 volunteers found that users are more likely to use neutral search terms (19).

In summary, the main findings of this study are the marked differences in the visibility of websites with a negative stance on vaccines given by the ranking by Google across not only different languages but also in different localized searches in English. Public health authorities, particularly those acting internationally, will need to take these differences into account when designing websites aiming at promoting vaccinations. They will also need to consider the relevance that issues like the adjuvants included in vaccine preparation have in the information available and clarify these issues to correct misinformation. Counteracting disinformation about vaccines by health authorities is part of the solution, but the loss of confidence in vaccines goes far beyond misinformation. Communities, social environment, educational level, are few examples of factors affecting the vaccine confidence. Education, as well as transparency, would be an important aspect to keep in mind when trying to increase vaccine confidence.

## Author Contributions

NA, PG, and MN designed the study. NA, MA-J, IB, GP, KC, MM, MN, and PG performed research. All authors analyzed data, all authors wrote the paper.

## Conflict of Interest Statement

The authors declare that the research was conducted in the absence of any commercial or financial relationships that could be construed as a potential conflict of interest.
