# Corrigendum: Fake News or Weak Science? Visibility and Characterization of Antivaccine Webpages Returned by Google in Different Languages and Countries

**DOI:** 10.3389/fimmu.2019.02252

**Published:** 2019-10-01

**Authors:** Nadia Arif, Majed Al-Jefri, Isabella Harb Bizzi, Gianni Boitano Perano, Michel Goldman, Inam Haq, Kee Leng Chua, Manuela Mengozzi, Marie Neunez, Helen Smith, Pietro Ghezzi

**Affiliations:** ^1^Brighton and Sussex Medical School, Falmer, United Kingdom; ^2^School of Computing, Engineering and Mathematics, University of Brighton, Brighton, United Kingdom; ^3^Universidade Federal do Rio Grande do Sul, Porto Alegre, Brazil; ^4^Sydney Medical School, University of Sydney, Sydney, NSW, Australia; ^5^Institute for Interdisciplinary Innovation in Healthcare, Université libre de Bruxelles, Brussels, Belgium; ^6^Lee Kong Chian School of Medicine, Nanyang Technological University, Singapore, Singapore

**Keywords:** information quality, google, Internet, news, news media, vaccines, autism, public understanding of science

In the original article, there was a mistake in **Supplementary Material Data Sheet 1** as published. In the tab “Australia” the value of cell M191 should be “0” instead of “1” and that of cell N191 should be “1” instead of “0.” The corrected **Supplementary Material Data Sheet 1** has been replaced in the original article.

In addition, there was a mistake in Table 6 as published. The percentage of vaccine-negative websites for Australia should be “33.3%” instead of “16.7%.” The corrected Table 6 appears below.

**Table 6:** Frequency of vaccine-negative webpages in each typology.

	**google.com**	**UK**	**AUS**	**FR**	**IT**	**Man**	**Port**	**Ara**
Comm	71.4	60.0	33.3	77.8	0.0	25.0	16.7	0.0
Gov	0.0	0.0	0.0	0.0	0.0	0.0	0.0	0.0
HP	0.0	0.0	16.7	0.0	12.5	27.8	7.1	0.0
News	6.8	7.0	2.0	0.0	2.1	12.1	7.3	3.9
NP	10.0	11.5	4.8	30.0	0.0	15.4	33.3	0.0
Other	34.0	40.0	39.7	30.8	30.6	29.8	56.4	15.8
Prof	7.7	7.7	5.0	7.1	0.0	10.0	17.4	0.0
ScJ	0.0	23.1	7.7	0.0	0.0	0.0	0.0	0.0
**Average vaccine-negative in the total search engine result page (SERP)**								
	16.6	19.7	16.0	19.6	11.5	19.0	24.2	7.5

*Data indicate the percentage of vaccine-negative webpages in each typology. Cells are color coded to show difference from “expected” based on the frequency in the total SERP shown in the bottom row (red, above the expected frequency; green, below the expected frequency)*.

The authors apologize for this error and state that this does not change the scientific conclusions of the article in any way. The original article has been updated.

